# The Path to an Evidence-Based Treatment Protocol for Extraoral Photobiomodulation Therapy for the Prevention of Oral Mucositis

**DOI:** 10.3389/froh.2021.689386

**Published:** 2021-07-16

**Authors:** Ather Adnan, Anna N. Yaroslavsky, James D. Carroll, Wayne Selting, Amy F. Juliano, Wendy B. London, Stephen T. Sonis, Christine N. Duncan, Nathaniel S. Treister

**Affiliations:** ^1^Texas A&M University Health Science Center, College of Medicine, Houston, TX, United States; ^2^Advanced Biophotonics Laboratory, Department of Physics and Applied Physics, University of Massachusetts Lowell, Lowell, MA, United States; ^3^Department of Dermatology, Massachusetts General Hospital, Boston, MA, United States; ^4^THOR Photomedicine, Chesham, United Kingdom; ^5^Department of Surgical Science and Integrated Diagnostics, University of Genoa, Genoa, Italy; ^6^Department of Radiology, Massachusetts Eye and Ear, Harvard Medical School, Boston, MA, United States; ^7^Department of Pediatrics, Dana-Farber/Boston Children's Cancer and Blood Disorders Center, Harvard Medical School, Boston, MA, United States; ^8^Department of Surgery, Divisions of Oral Medicine and Dentistry, Brigham and Women's Hospital and the Dana-Farber Cancer Institute, Boston, MA, United States; ^9^Department of Oral Medicine, Infection and Immunity, Harvard School of Dental Medicine, Boston, MA, United States; ^10^Biomodels LLC., Waltham, MA, United States

**Keywords:** photobiomodulation therapy, oral mucositis, low level light therapy, hematopoietic stem cell transplant, monte carlo

## Abstract

Oral mucositis is a painful complication of hematopoietic stem cell transplantation for which photobiomodulation therapy (PBMT) is a safe and effective intervention. Extraoral delivery of PBMT has clinical advantages over intraoral delivery but requires additional dosimetric considerations due to the external tissue layers through which the light must propagate before reaching the oral mucosa. Additionally, to date there has been no dose modeling study, a task essential to developing a justified treatment protocol. We review here some of the complexities surrounding extraoral photobiomodulation therapy and offer that may help guide researchers toward an evidence-based treatment protocol for the prevention of oral mucositis.

## Introduction

**Figure 1:**
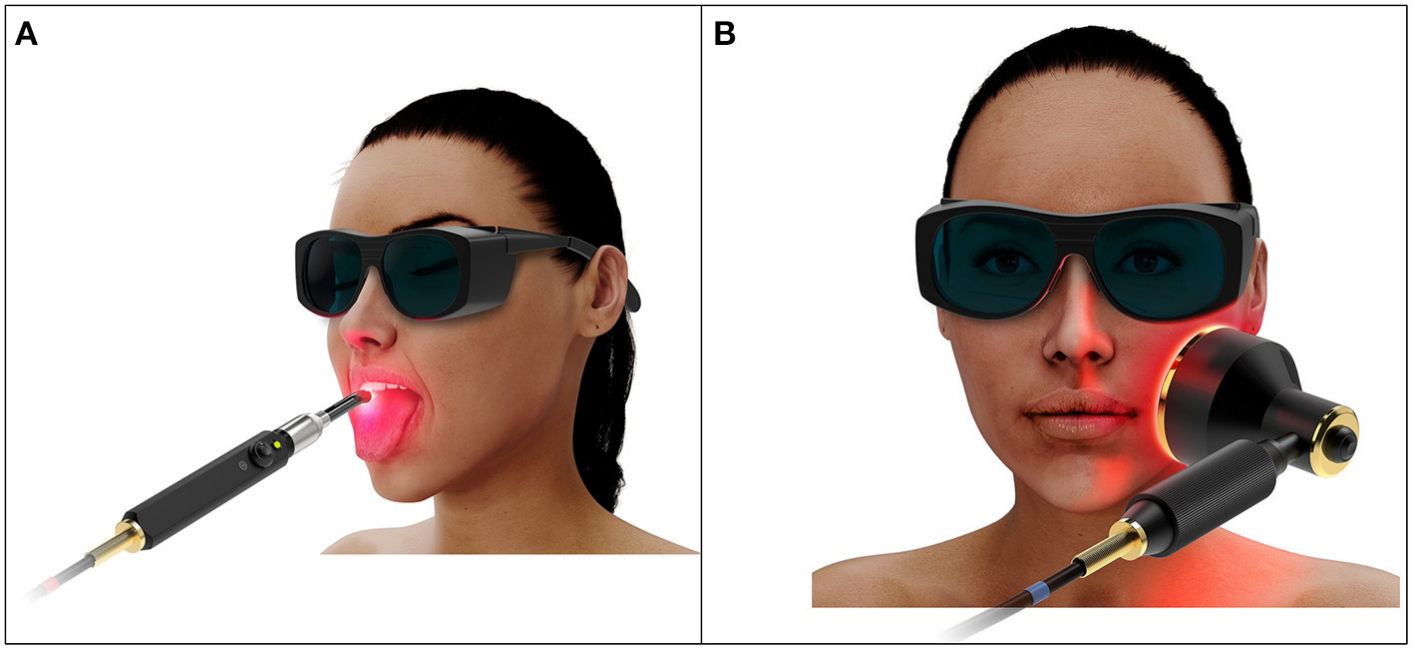
Artistic representation of **(A)** intraoral and **(B)** extraoral photobiomodulation therapy.

Oral mucositis (OM) is a painful complication of hematopoietic stem cell transplantation (HSCT) characterized by inflammation and ulceration of the oral mucosa [1]. Photobiomodulation therapy (PBMT) is a safe and effective light-based intervention that has been shown to prevent and treat OM in HSCT patients [2, 3]. Current recommended PBMT protocols utilize intraoral delivery that involves multiple sequential dose administrations in a spot-by-spot manner, an approach that is technically complex and time consuming, and that requires a high level of patient cooperation (Figure 1A) [4].

Extraoral PBMT is likely to be clinically advantageous as its application is simpler and its treatment fields are more likely to include distal mucosae that are not reached by intraoral delivery. However, extraoral delivery requires transport of photons through the external orofacial tissue layers such as skin, fat, and muscle before reaching the inner mucosal lining, attenuating the dose delivered and requiring complex dosimetric considerations (Figure 1B). Additionally, no dosimetric study or justified protocol has been reported. The purpose of this review is to carefully consider the complexities of extraorally delivered PBMT and work toward development of an evidence-based treatment protocol for OM prevention.

## Challenges Surrounding Extraoral PBMT for OM

Intraoral PBMT is delivered directly to the mucosal surface, targeting the underlying connective tissue at an approximate depth of 100–700 μm [5]. The challenges introduced with an extraoral delivery of PBMT result from the additional layers of tissue through which the light must propagate in order to reach the oral mucosa. These layers are optically thick and attenuate the dose delivered. We review here the basic anatomy of the orofacial tissues and discuss some of the salient consequences from a photobiological standpoint.

### Layers of the Orofacial Tissues

**Figure 2:**
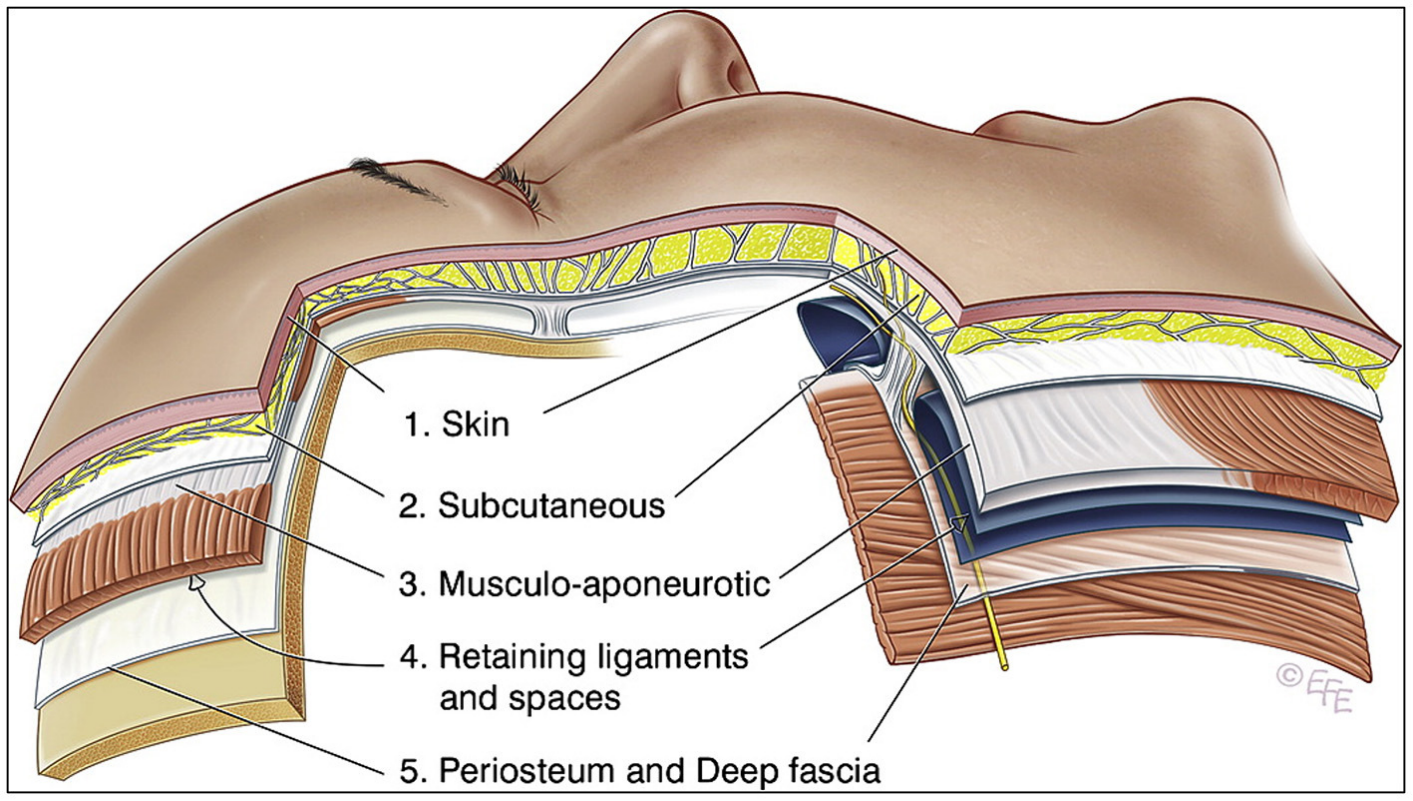
Layer model diagram of facial tissues. Adapted with permission from Mendelson et al.

The tissues of the scalp and face are frequently simplified into five layers, from superficial to deep: (1) the skin, (2) the subcutaneous layer, (3) the musculoaponeurotic layer, (4) the spaces and retaining ligament, and (5) the deep fascia (Figure 2) [6]. Bone and periosteum are not relevant as they are avoidable during the delivery of extraoral PBMT and would otherwise cause additional dose attenuation. From a photobiological perspective, the orofacial tissue layers can be simplified into skin, fat, and muscle. Each of these layers exhibit different optical properties. Out of the three tissues, skin is the most attenuating layer and responsible for most of the absorption and scattering of light due to the chromophore melanin (more specifically eumelanin, but for simplicity will be referred to more generally as melanin) [7, 8]. Darker skin has a higher concentration of melanin and thus is more attenuating.

### Degree of Attenuation

The degree of attenuation by skin, and to a lesser extent the subdermal tissues, is significant. To illustrate, even in skin types of low melanin concentration, light at a wavelength of 600 nm is attenuated to 37% of its incident power at a depth of only 550 μm from the skin surface; increasing the wavelength to 800 nm increases the depth to 1,200 μm [9]. A study of optical properties of human tissues reported a scattering coefficient of 2.73 mm^−1^ at a wavelength of 633 nm in dermis of low melanin concentration, decreasing to 1.63 mm^−1^ at a wavelength of 900 nm. Absorption and scattering coefficients of the subdermal tissues (fat and muscle) were found to be lower though still significant [7]. The average thickness of the human cheek is on the order of 6–7 millimeters [10]. While only a proportion of this is skin, the implication is that a large percentage of the incident power is lost while passing through the various tissues before reaching the oral mucosa. This has important consequences on treatment duration. For example, a 90% reduction in dose transmission would require a 10-fold increase in treatment duration to deliver the same dose to the oral mucosa. Maximizing penetration is therefore advantageous from a protocol feasibility standpoint and, as demonstrated, increasing wavelength decreases the magnitude of scattering and absorption by tissues and is a method to achieve this.

### Variability of Attenuation

Variability among patients related to anatomical differences and skin type contributes to differences in the dose transmitted to the oral mucosa. This variability is unpredictable and does not reliably correlate with sex or age. For example, a study of ultrasonographic measurements of the cheek in 30 adults aged 24–61 years revealed an average cheek dermis thickness of 1,639.27 μm with a relatively large standard deviation of 531.53 μm [11]. There were no apparent differences by sex or age, suggesting that splitting patients into groups would not help address this variance.

Skin types of higher Fitzpatrick score, a numerical classification of the color and tanning ability of skin, contain higher concentrations of melanin and thus are more attenuating [7]. In effect, patients with a skin type of higher melanin concentration would receive a lower transmitted dose to the oral mucosa despite receiving the same applied dose. Of note, the difference in attenuation is lessened at longer wavelengths. One study of *ex vivo* dermal samples obtained from subjects with skin types of lower vs. higher melanin concentration reported reduced scattering coefficients of 2.73 ± 0.54 mm^−1^ vs. 3.21 ± 2.04 mm^−1^ at 633 nm compared to 1.63 ± 0.25 mm^−1^ vs. 1.81 ± 0.040 mm^−1^ at 900 nm [7]. Two additional studies of the optical properties of skin *in vivo* that included patients of Fitzpatrick skin types I–VI similarly found higher absorption coefficients in higher Fitzpatrick skin types. This difference decreased in magnitude across the wavelength range of 600–800 nm, and at 850 nm there was no significant difference in absorption coefficients [12, 13]. Furthermore, skin pigmentation was found to have a greater influence on reflection at wavelengths of 460–700 nm compared to 800–850 nm [14, 15]. These findings suggest that a longer wavelength would help minimize differences in dose delivery based on skin type, as well as increase penetration overall.

### Safety and Feasibility

There has been no reported toxicity in any of the studies of PBMT for the prevention and/or management of OM [16]. In the limited number of studies evaluating extraoral PBMT, there has similarly been no reported cutaneous or oral toxicity. In theory, PBMT could lead to heating of tissues and, when applied extraorally, heating of the skin; however, the American National Standards Institute (ANSI) publishes safety standards which establish the maximal permissible exposure (MPE) for skin exposure (applies to all skin types) to a laser beam [17], which can serve as a reference guideline. A study that included patients with skin of and device parameters within ANSI standards investigated the effects of melanin on skin surface temperature when exposed to PBMT. The authors reported no significant skin temperature differences with doses ranging from 0 J to 50 J via concurrent use of super-pulsed lasers and pulsed red and infrared LEDs at wavelengths of 810–904 nm [18].

There have been two studies investigating the feasibility of extraoral PBMT treatments in an inpatient pediatric hematology-oncology unit. Both met goal endpoints in feasibility, tolerance, and safety of the intervention. The first study enrolled 10 patients aged 4 to 21 years and reported successful administration of prophylactic daily extraoral PBMT in 347/355 (97.7%) sessions by 10 trained nurses with no pain or other reason to discontinue therapy [19]. The second study employed a curative (not prophylactic) mixed intraoral/extraoral PBMT protocol that enrolled 22 patients aged 3 to 18 years with WHO Grade ≥2 OM, and reported procedural success (administration of PBMT to entire surface of oral mucosa at least 3 times every 2 days in first 7 days of OM) in 77% of episodes. Treatments were well-tolerated and there were no treatment-related adverse events [20].

### Summary

These findings taken together guide our approach to developing a treatment protocol for extraoral delivery of PBMT. First, because of the significant degree of attenuation caused by the orofacial skin and tissues, the treatment protocol should aim to maximize penetration lest the treatment time required to achieve an efficacious dose would be infeasible. Second, the same regimen administered to two patients will likely result in two slightly different doses transmitted to the oral mucosa, necessitating a standardized protocol that aims to treat the “median” patient, akin to pharmacological agents with standard dosing despite variable pharmacokinetics and pharmacodynamics.

## Treatment Protocol for Extraoral PBMT

Currently, there is no established treatment protocol for extraoral PBMT for prevention of OM. To our knowledge, no rigorous dosimetric study of extraorally delivered PBMT estimating the dose transmitted to the mucosal surface has been performed. To date, five clinical studies have been reported that investigate the efficacy of extraoral PBMT for OM [21–25]. None provide an estimated dose transmitted to the oral mucosa by the treatment protocol used. In the first four studies, wavelengths (660–680 nm) and irradiance (50–100 mW), with the exception of two studies which additionally had an 830 nm study arm, were similar to those utilized for intraoral delivery [21–24]; however, as described earlier, this does not result in the same dose delivered to oral mucosa due to attenuation from external tissue layers. The fifth study, which compared intraoral and extraoral PBMT, utilized a higher irradiance of 407 mW/cm^2^ in the extraoral arm, delivering 4 J/cm^2^ over 10 s at six different locations. A dual wavelength 810/980 nm device was used [25]. While this protocol likely delivered a dose closer to the therapeutic range (1–6 J/cm^2^) than the preceding studies, the exact dose is still unknown; to transmit a dose of at least 1 J/cm^2^ with this protocol would require a percent transmission of 25%, which is likely higher than the true penetration of infrared/near-infrared light through the average human cheek. The two aforementioned feasibility studies utilized devices with two wavelengths, one red (635–660 nm) and one near infrared (815–830 nm) [19, 20]. One study applied 50 mW/cm^2^ to 6 sites for 1 min each [19]. The other employed a scanning approach, applying 4 W/cm^2^ with a laser fiber across the external cheek, for 1 second per cm^2^, as well as some intraoral application at a lower irradiance [20]. The first protocol likely did not reach the target dose delivered to the mucosa and the second exceeded ANSI safety recommendations. One study of extraoral PBMT for OM in rats has been reported [26]. The study used a dual wavelength 810/980 nm device and applied 407 mW/cm^2^ for 15 or 30 s. However, rat orofacial anatomy is different from human anatomy and the percent dose transmitted during extraoral PBMT delivery in rats is not the same as in humans. In nearly all cases, justification for the selection of device parameters has been attributed to prior intraoral or limited extraoral studies rather than an approach based on orofacial anatomy and photobiological principles. The following is our approach to a rational and scientifically based treatment protocol in the context of the previously considered challenges surrounding extraoral PBMT delivery to the oral mucosa.

### Target Dose

Intraoral PBMT OM prevention protocols recommend a target dose on the order of 1.0–6.2 J/cm^2^, although the true therapeutic range may be broader [4]. Given that extraoral PBMT acts by the same mechanism, the target dose should be the same. However, there are a few considerations to be made. First, as explained previously there is unavoidable variability in the dose delivered to the oral mucosa due to variation in orofacial anatomy. Thus, with a standardized protocol that treats the “median” patient, there will be some degree of under- and overdosing. Given the relatively broad range of effective dose, the transmitted dose should still have a therapeutic effect, particularly if a middling target dose is selected [27]. Second, a potential limiting factor of extraoral PBMT is the long treatment duration required to deliver the total target dose. Consequently, a very high target dose should be avoided, in order to afford a more feasible treatment duration, and the rate of dose delivery should be optimized by maximizing penetration (i.e., wavelength) and power output. Third, in regards to safety, no surface skin temperature changes were observed in volunteers of varying skin type exposed to PBMT at wavelengths of 640, 875, and 904 nm and energy of up to 50 J, an order of magnitude above the usual dose indicated for PBMT for OM [18]. Thus, the degree of under- or overdosing caused by anatomical or skin type variation is likely insufficient to warrant safety concerns.

### Wavelength

Intraoral protocols utilize wavelengths in the red light range: 632.8 nm for He-Ne lasers and 660 nm for diode lasers [4]. While this range is appropriate for superficial treatment, as in the case of direct application to the oral mucosa, there are many reasons to utilize the longest wavelength with evidence of efficacy as mentioned earlier: (1) there is decreased absorption and scattering of light by melanin, fat, and muscle at longer wavelengths allowing for increased dose delivery and thus a more feasible treatment duration, and (2) variation in dose attenuation due to the effects of melanin is lessened at longer wavelengths [7]. There is both preclinical and clinical evidence of efficacy for longer wavelength PBMT for OM. Cytochrome oxidase C, an important chromophore thought to mediate the therapeutic effects of PBMT, holds activity “peaks” or “hotspots” suggesting bioequivalency throughout these peaks rather than at any one wavelength [28]. The highest peak is in the near infrared window at 812.0–846 nm. Additionally, PBMT in the near infrared window has shown efficacy for several other inflammatory/painful indications, such as osteoarthritis, colitis, and temporomandibular disorders [29–36]. Longer wavelengths beyond the near-infrared range lack evidence of efficacy [37].

### Irradiance

Irradiance seems to be less significant than fluence with regard to efficacy and can be manipulated to afford a feasible treatment duration. Indeed, intraoral protocols utilize a broad range of irradiances, 24–31.25 mW/cm^2^ for He-Ne lasers and 417–1,000 mW/cm^2^ for diode lasers [4]. In keeping with the goal of maximizing dose delivery rate, the irradiance should be maximized while in accordance with ANSI standards. This helps attain a feasible treatment duration while minimizing any safety concerns.

### Treatment Sites and Duration

The mucosal surfaces of the oral cavity to consider include the buccal mucosa, upper and lower lip, ventral tongue, lateral tongue, floor of mouth, and soft palate. Mucosae distal to this potentially reachable by an extraoral approach include the oropharyngeal and esophageal mucosa, a concept further supported by studies indicating locoregional or even systemic therapeutic effects [38, 39]. An extraoral protocol should aim to treat all these sites with minimal overlap and avoidance of teeth, bone, and cartilage to decrease dose attenuation (Table 1). This approach assumes the oral mucosa itself is very thin and inconsequential in terms of dose attenuation, and that the small amount of air contained within the oral cavity is similarly optically negligible. As a result, the trajectories treating the buccal mucosa should also reach and treat the lateral tongue and soft palate, and those treating the floor of mouth should also reach and treat the ventral tongue.

**Table 1:** Proposed protocol of treatment locations and trajectories and their target mucosal surface for use in extraoral delivery of photobiomodulation therapy for prevention of oral mucositis.

**Treatment location and trajectory**	**Mucosal surface treated**
Left cheek, transversely	Left buccal mucosa and lateral tongue
Right cheek, transversely	Right buccal mucosa and lateral tongue
Philtrum, anteroposteriorly	Upper lip and lower lip
Midline neck, vertically	Midline floor of mouth, ventral tongue, oropharyngeal mucosa, and esophageal mucosa
Left neck, transversely	Left floor of mouth, ventral tongue, oropharyngeal mucosa, and esophageal mucosa
Right neck, transversely	Right floor of mouth, ventral tongue, oropharyngeal mucosa, and esophageal mucosa

The treatment duration should aim to deliver the target therapeutic dose and is dependent on the rate of dose delivery (energy fluence rate, J/cm^2^/s) to the oral mucosa and varies by treatment site. This parameter can only be determined after a rigorous dosimetric study investigating the degree of attenuation of PBMT by orofacial structures along each treatment site trajectory. Due to dose attenuation, it is likely that the treatment duration required for extraoral PBMT will be considerably longer than that required by intraoral PBMT; however, device design can allow for simple and comfortable handsfree delivery. Important practical aspects of the delivery of extraoral PBMT, including device design and handling, will be essential to optimizing efficiency of delivery, for example by allowing delivery to multiple treatment sites concurrently.

### Future Directions

There are a few important barriers to implementation of extraoral PBMT for OM. First, to date there has been no reported dose modeling of extraoral PBMT, information which would be essential to inform the creation of a justified, validated treatment protocol. Critical aspects of this dosimetric study include the determination of the “median” patient in terms of orofacial morphology, the modeling of dose transmission to the oral mucosa along several treatment trajectories given a set of treatment parameters, and an *in vivo* validation of these findings. Second, the treatment protocol would need to be evaluated for efficacy in a randomized, placebo-controlled clinical trial evaluating outcomes such as incidence and duration of severe OM.

## Conclusions

Intraoral PBMT is a safe and effective treatment for OM among patients receiving cytotoxic conditioning regimens prior to HSCT. Extraoral PBMT has advantages over intraoral PBMT but lacks evidence of efficacy and requires additional dosimetric considerations due to the anatomical structures the light must pass through before reaching the oral mucosa. Thus, the device parameters used in intraoral PBMT are not appropriate for extraoral PBMT. While it is evident that treatment duration needs to be longer for extraoral PBMT than intraoral PBMT, measures can be applied to minimize treatment time and optimize ease and comfort of delivery. We have outlined the necessary steps to establish and validate a justified treatment protocol that can be evaluated for efficacy in a randomized clinical trial and ultimately used in clinical practice.

## Author Contributions

All authors listed have made a substantial, direct and intellectual contribution to the work, and approved it for publication.

## Conflict of Interest

JC is CEO of THOR Photomedicine. WL receives payment for service on Data and Safety Monitoring Boards for Merck and Jubilant Draximage. SS reports personal fees from Biomodels, LLC, personal fees from Primary Endpoint Solutions, LLC, outside of the submitted work. As an employee of Biomodels and PES, he is involved in assisting industry, government and academics in the study and enablement of drugs, biologicals and devices to treat patients for a broad range of indications including cancer and oral toxicities of cancer therapy. He does not have equity or receive payment from any of the companies' clients. NT serves as a consultant for MuReva Phototherapy Inc. The remaining authors declare that the research was conducted in the absence of any commercial or financial relationships that could be construed as a potential conflict of interest.

## References

[1] Sonis ST. The pathobiology of mucositis. Nat Rev Cancer. (2004) 4:277–84. 10.1038/nrc1318 15057287

[2] Oberoi S Zamperlini-Netto G Beyene J Treister NS Sung L. Effect of prophylactic low level laser therapy on oral mucositis: a systematic review and meta-analysis. PLoS ONE. (2014) 9:e107418. 10.1371/journal.pone.0107418 25198431 PMC4157876

[3] Miranda-Silva W Gomes-Silva W Zadik Y Yarom N Al-Azri AR Hong CHL. MASCC/ISOO clinical practice guidelines for the management of mucositis: sub-analysis of current interventions for the management of oral mucositis in pediatric cancer patients. Sup Care Cancer. (2020) 29:3539–62. 10.1007/s00520-020-05803-4 33156403

[4] Zadik Y Arany PR Fregnani ER Bossi P Antunes HS Bensadoun RJ. Systematic review of photobiomodulation for the management of oral mucositis in cancer patients and clinical practice guidelines. Sup Care Cancer. (2019) 27:3969–83. 10.1007/s00520-019-04890-2 31286228

[5] Stasio DD Lauritano D Iquebal H Romano A Gentile E Lucchese A. Measurement of oral epithelial thickness by optical coherence tomography. Diagnostics. (2019) 9:90. 10.3390/diagnostics9030090 31390841 PMC6787684

[6] Mendelson BC Jacobson SR. Surgical anatomy of the midcheek: facial layers, spaces, and the midcheek segments. Clin Plast Surg. (2008) 35:395–404; discussion 393. 10.1016/j.cps.2008.02.003 18558234

[7] Simpson CR Kohl M Essenpreis M Cope M. Near-infrared optical properties of ex vivo human skin and subcutaneous tissues measured using the monte carlo inversion technique. Phys Med Biol. (1998) 43:2465–78. 10.1088/0031-9155/43/9/003 9755939

[8] Simon JD Peles DN. The red and the black. Acc Chem Res. (2010) 43:1452–60. 10.1021/ar100079y 20734991

[9] Anderson RR Parrish JA. The optics of human skin. J Invest Dermatol. (1981) 77:13–9. 10.1111/1523-1747.ep12479191 7252245

[10] Kim YS Lee KW Kim JS Gil YC Tanvaa T Shin DH. Regional thickness of facial skin and superficial fat: application to the minimally invasive procedures. Clin Anat. (2019) 32:1008–18. 10.1002/ca.23331 30629772

[11] Firooz A Rajabi-Estarabadi A Zartab H Pazhohi N Fanian F Janani L. The influence of gender and age on the thickness and echo-density of skin. Skin Res Technol. (2017) 23:13–20. 10.1111/srt.12294 27273751

[12] Tseng SH Grant A Durkin AJ. *In vivo* determination of skin near-infrared optical properties using diffuse optical spectroscopy. J Biomed Opt. (2008) 13:014016. 10.1117/1.2829772 18315374 PMC2626348

[13] Tseng SH Bargo P Durkin A Kollias N. Chromophore concentrations, absorption and scattering properties of human skin *in-vivo*. Opt Express. (2009) 17:14599–617. 10.1364/OE.17.014599 19687939 PMC2754563

[14] Zonios G Bykowski J Kollias N. Skin melanin, hemoglobin, and light scattering properties can be quantitatively assessed *in vivo* using diffuse reflectance spectroscopy. J Invest Dermatol. (2001) 117:1452–7. 10.1046/j.0022-202x.2001.01577.x 11886508

[15] Sampson DD Murphy BW. How can optics be used to sense skin cancer? In: [Conference presentation]. SPIE 5855, 17th International Conference on Optical Fibre Sensors (Bruges) (2005). 10.1117/12.623383

[16] Bensadoun RJ Epstein JB Nair RG Barasch A Raber-Durlacher JE Migliorati C. Safety and efficacy of photobiomodulation therapy in oncology: a systematic review. Cancer Med. (2020) 9:8279–300. 10.1002/cam4.3582 33107198 PMC7666741

[17] ANSI. American National Standard for Safe Use of Lasers. Orlando, FL: Laser Institute of America (2007).

[18] Grandinetti Vdos S Miranda EF Johnson DS de Paiva PR Tomazoni SS Vanin AA. The thermal impact of phototherapy with concurrent super-pulsed lasers and red and infrared LEDs on human skin. Lasers Med Sci. (2015) 30:1575–81. 10.1007/s10103-015-1755-0 25987340

[19] Treister NS London WB Guo D Malsch M Verrill K Brewer J. A feasibility study evaluating extraoral photobiomodulation therapy for prevention of mucositis in pediatric hematopoietic cell transplantation. Photomed Laser Surg. (2016) 34:178–84. 10.1089/pho.2015.4021 26982624

[20] Noirrit-Esclassan E Valera MC Vignes E Munzer C Bonal S Daries M. Photobiomodulation with a combination of two wavelengths in the treatment of oral mucositis in children: the PEDIALASE feasibility study. Arch Pediatr. (2019) 26:268–74. 10.1016/j.arcped.2019.05.012 31281038

[21] Moraes JJC Queiroga AS De Biase RCCG Leite EP Cabral Júnior CR Limeira Júnior FA. The effect of low level laser therapy in different wavelengths in the treatment of oral mucositis—proposal for extra-oral implementation. Laser Physics. (2009) 19:1912–9. 10.1134/S1054660X09170150

[22] Whelan HT Connelly JF Hodgson BD Barbeau L Post AC Bullard G. NASA light-emitting diodes for the prevention of oral mucositis in pediatric bone marrow transplant patients. J Clin Laser Med Surg. (2002) 20:319–24. 10.1089/104454702320901107 12513918

[23] Hodgson BD Margolis DM Salzman DE Eastwood D Tarima S Williams LD. Amelioration of oral mucositis pain by NASA near-infrared light-emitting diodes in bone marrow transplant patients. Sup Care Cancer. (2012) 20:1405–15. 10.1007/s00520-011-1223-8 21725826

[24] Soto M Lalla RV Gouveia RV Zecchin VG Seber A Lopes NN. Pilot study on the efficacy of combined intraoral and extraoral low-level laser therapy for prevention of oral mucositis in pediatric patients undergoing hematopoietic stem cell transplantation. Photomed Laser Surg. (2015) 33:540–6. 10.1089/pho.2015.3954 26501372

[25] Ramos-Pinto MB de Lima Gusmao TP Schmidt-Filho J Jaguar GC Martins MD Alves FA. Intraoral versus extraoral photobiomodulation therapy in the prevention of oral mucositis in HSCT patients: a randomized, single-blind, controlled clinical trial. Sup Care Cancer. (2021). 10.1007/s00520-021-06228-3. [Epub ahead of print]. 33905011

[26] Thieme S Ribeiro JT Dos Santos BG de Almeida Zieger R Severo MLB Martins MAT. Comparison of photobiomodulation using either an intraoral or an extraoral laser on oral mucositis induced by chemotherapy in rats. Supp Care Cancer. (2020) 28:867–76. 10.1007/s00520-019-04889-9 31165336

[27] Huang YY Sharma SK Carroll J Hamblin MR. Biphasic dose response in low level light therapy - an update. Dose Resp. (2011) 9:602–18. 10.2203/dose-response.11-009.Hamblin 22461763 PMC3315174

[28] Karu TI Kolyakov SF. Exact action spectra for cellular responses relevant to phototherapy. Photomed Laser Surg. (2005) 23:355–61. 10.1089/pho.2005.23.355 16144476

[29] Alghadir A Omar MT Al-Askar AB Al-Muteri NK. Effect of low-level laser therapy in patients with chronic knee osteoarthritis: a single-blinded randomized clinical study. Lasers Med Sci. (2014) 29:749–55. 10.1007/s10103-013-1393-3 23912778

[30] Zigmond E Varol C Kaplan M Shapira O Melzer E. Low-level light therapy induces mucosal healing in a murine model of dextran-sodium-sulfate induced colitis. Photomed Laser Surg. (2014) 32:450–7. 10.1089/pho.2013.3626 25101535

[31] de Castro IC Rosa CB Carvalho CM Aragao JS Cangussu MC Dos Santos JN. Assessment of different energy delivery settings in laser and LED phototherapies in the inflammatory process of rat's TMJ induced by carrageenan. Lasers Med Sci. (2015) 30:2105–13. 10.1007/s10103-015-1748-z 25854994

[32] Panhoca VH Lizarelli Rde F Nunez SC Pizzo RC Grecco C Paolillo FR. Comparative clinical study of light analgesic effect on temporomandibular disorder (TMD) using red and infrared led therapy. Lasers Med Sci. (2015) 30:815–22. 10.1007/s10103-013-1444-9 24197518

[33] Ferraresi C Parizotto NA Pires de Sousa MV Kaippert B Huang YY Koiso T. Light-emitting diode therapy in exercise-trained mice increases muscle performance, cytochrome c oxidase activity, ATP and cell proliferation. J Biophotonics. (2016) 9:976. 10.1002/jbio.201680087 27592534

[34] Silveira PC Ferreira KB da Rocha FR Pieri BL Pedroso GS De Souza CT. Effect of low-power laser (LPL) and light-emitting diode (LED) on inflammatory response in burn wound healing. Inflammation. (2016) 39:1395–404. 10.1007/s10753-016-0371-x 27206919

[35] Frangez I Cankar K Ban Frangez H Smrke DM. The effect of LED on blood microcirculation during chronic wound healing in diabetic and non-diabetic patients-a prospective, double-blind randomized study. Lasers Med Sci. (2017) 32:887–94. 10.1007/s10103-017-2189-7 28342007

[36] Tuner J Hosseinpour S Fekrazad R. Photobiomodulation in Temporomandibular disorders. Photobiomodul Photomed Laser Surg. (2019) 37:826–36. 10.1089/photob.2019.4705 31770071

[37] Chung H Dai T Sharma SK Huang YY Carroll JD Hamblin MR. The nuts and bolts of low-level laser (light) therapy. Ann Biomed Eng. (2012) 40:516–33. 10.1007/s10439-011-0454-7 22045511 PMC3288797

[38] Hopkins JT McLoda TA Seegmiller JG David Baxter G. Low-level laser therapy facilitates superficial wound healing in humans: a triple-blind, sham-controlled study. J Athl Train. (2004) 39:223–9. 15496990 PMC522143

[39] Braverman B McCarthy RJ Ivankovich AD Forde DE Overfield M Bapna MS. Effect of helium-neon and infrared laser irradiation on wound healing in rabbits. Lasers Surg Med. (1989) 9:50–8. 10.1002/lsm.1900090111 2927230

